# Experience from a Fast-Track Multidisciplinary Clinic Integrating Movement Disorders Neurologists in Normal Pressure Hydrocephalus Evaluation

**DOI:** 10.3390/jcm13206135

**Published:** 2024-10-15

**Authors:** Saud Alhusaini, Kathryn Sine, Prarthana Prakash, Laura E. Korthauer, Seth A. Margolis, Andrew Chen, Nicole Rawnsley, Elizabeth Breen, Kenneth Vinacco, Emily Weisbach, Maria Guglielmo, Umer Akbar, Jennifer D. Davis, Konstantina Svokos, Petra Klinge

**Affiliations:** 1 Department of Neurology, Alpert Medical School of Brown University, Providence, RI 02903, USA; kathryn_sine@brown.edu (K.S.); prarthana_prakash@brown.edu (P.P.); andrew_l_chen@brown.edu (A.C.); emily_weisbach@brown.edu (E.W.); umer_akbar@brown.edu (U.A.); 2 The Movement Disorders Program, Rhode Island Hospital, Providence, RI 02903, USA; 3 Neuropsychology Program, Rhode Island Hospital, Providence, RI 02903, USA; laura_korthauer@brown.edu (L.E.K.); seth_margolis@brown.edu (S.A.M.); jennifer_davis@brown.edu (J.D.D.); 4 Department of Psychiatry & Human Behavior, Alpert Medical School of Brown University, Providence, RI 02903, USA; 5 Rehabilitation Services, Rhode Island Hospital, Providence, RI 02903, USA; nicole.rawnsley@lifespan.org (N.R.); ebreen@lifespan.org (E.B.); kvinacco@lifespan.org (K.V.); 6 Neurosurgery Department, Rhode Island Hospital and Alpert Medical School of Brown University, Providence, RI 02903, USA; maria_guglielmo@brown.edu (M.G.); konstantina_svokos@brown.edu (K.S.); petra_klinge@brown.edu (P.K.)

**Keywords:** NPH, multidisciplinary, movement disorders neurologists, shunting

## Abstract

In this prospective observational cohort study, we provide preliminary findings from a same-day multidisciplinary fast-tracked normal pressure hydrocephalus (NPH) clinic; incorporating the expertise of movement disorders neurologists, emphasizing the clinical characteristics, consensus classification, and management of patients referred for suspected NPH. We evaluated 111 patients (male/female: 67/44) from April 2022 to May 2023. Based on the multidisciplinary team consensus, 52 (46.8%) were classified as “probable” idiopathic NPH (iNPH), 14 (12.6%) as “possible” NPH, 42 (37.8%) as “unlikely” NPH, and three (2.7%) as secondary NPH. While parkinsonian syndromes were recognized in 19.2% of “probable” iNPH patients (vs. 7.1% in “possible” and 26.2% in “unlikely” NPH), no significant group differences were noted in the scores of the UPDRS-III scale. Degenerative spine pathologies were prevalent across all NPH categories, affecting at least 50% of patients. In the “probable” iNPH group, 78.8% received programmable ventriculoperitoneal shunts, with clinical improvement identified in 87.8% at 12-month follow-up. Our findings underscore the high prevalence of overlapping and competing movement and spinal disorders in patients with suspected NPH. Further, our novel approach, incorporating movement disorder neurologists in NPH multidisciplinary evaluation, improved diagnostic precision and streamlined personalized plans, including further neurological workups, necessary spinal interventions, and medical management or rehabilitation.

## 1. Introduction

After more than 5 decades of recognizing normal pressure hydrocephalus (NPH) as a clinical syndrome, it continues to face diagnostic challenges and broad differential diagnoses [1,2,3,4,5,6]. Given the considerably heterogeneous features shared between NPH and many other movement and neurodegenerative disorders (e.g., Alzheimer’s disease [AD], idiopathic Parkinson’s disease [PD], and other parkinsonian syndromes), a multidisciplinary team evaluation is often recommended [7,8,9,10,11]. This multidisciplinary approach ensures a comprehensive assessment of diverse clinical presentations, proper management planning, and accurate selection of patients for cerebrospinal fluid (CSF) shunting, specifically ventriculoperitoneal shunting (VPS).

In recent years, the focus of clinical research has shifted from examining the overlap between NPH and different movement disorders (e.g., idiopathic PD) to understanding the mechanisms linking NPH to dementia [12]. Despite the high prevalence of gait disturbance, imbalance, and parkinsonism in patients with clinical features suggestive of NPH, there is little data on the value of movement disorders expertise in the interdisciplinary evaluation of these patients [13,14]. For example, similar patterns of slow gait and imbalance were previously identified in patients with idiopathic PD and NPH, highlighting the necessity to differentiate NPH from common movement disorders. In early 2022, we began a dedicated 1-day fast-track multidisciplinary NPH clinic in conjunction with the departments of neurology (division of movement disorders), neurosurgery, neuropsychology, and rehabilitation services. Here, we present our first-year multidisciplinary experience incorporating the expertise of movement disorders neurologists, focusing on the clinical characteristics, risk factors, consensus diagnoses and classification, and initial management planning of patients referred for suspected NPH.

## 2. Methods

In this prospective observational cohort study, we evaluated patients referred to our multidisciplinary fast-track clinic with suspected NPH between April 2022 and May 2023 (Institutional IRB 1578948-9).

### 2.1. Clinical Evaluations

All patients were referred for suspected NPH due to the presence of ≥1 symptom of the NPH triad (gait disturbance, cognitive symptoms, and urinary incontinence) and evidence of cerebral ventriculomegaly on brain imaging. Patients were referred by various clinicians, including primary care physicians (PCPs), non-movement disorders neurologists (e.g., community-based general neurologists), neurosurgeons, emergency room (ER) physicians, and hospitalists. None of the patients received a prior workup or diagnosis of NPH.

**Figure 1:**
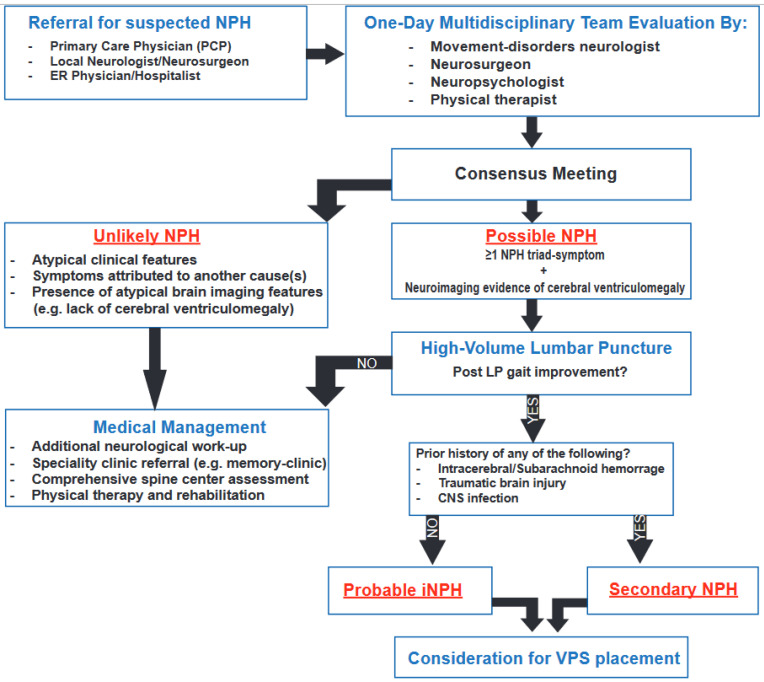
Pathway for Reaching Final NPH Classification. CNS: central nervous system; CSF: cerebrospinal fluid; LP: lumbar puncture; VPS: ventriculoperitoneal shunt.

Patients underwent a same-day comprehensive evaluation by a multidisciplinary team of movement-disorders neurologists, neurosurgeons, neuropsychologists, and physical therapists specialized in neurological conditions (see Figure 1). The in-clinic evaluation included collecting a detailed clinical history, complete physical and neurological examinations (including movement evaluations using the Unified Parkinson’s Disease Rating Scale–Motor Examination- part III, UPDRS-III) [15], neuropsychological assessments of cognition, mood, and other aspects of behavior (see Supplementary Material for a list of neurocognitive tests), gait and balance assessments, and interpretation of neuroimaging findings, particularly magnetic resonance imaging (MRI) of the brain. A neuroradiologist blind to the multidisciplinary team evaluations independently reviewed all brain imaging. Evan’s index > 0.3, in the absence of CSF flow obstruction, was the sole criterion mandated to establish the radiological finding of cerebral ventriculomegaly [16]. The degree of MRI periventricular white matter signal changes and the presence of focal brain atrophy were considered relevant and supported the decision-making as indicated (e.g., if there was a clinical concern for a co-morbid neurodegenerative or cerebrovascular disease). We also considered the NPH neuroimaging feature proposed by the Japanese Hydrocephalus Society: narrowing of the sulci and subarachnoid spaces over the high convexity and midline surface of the brain and enlargement of the Sylvian fissures and basal cisterns [often referred to as disproportionately enlarged subarachnoid space hydrocephalus (DESH)] as a supportive biomarker of “probable” idiopathic NPH (iNPH) [17,18].

### 2.2. Classification of Patients with Suspected NPH

As shown in Figure 1, patients were classified following the 2005 international iNPH guidelines as having [19]: (1) “unlikely” NPH (due to symptoms more likely explained by another co-morbid condition + the presence of atypical clinical [e.g., lack of NPH triad] or neuroimaging features); (2) “possible” NPH (due to presence of ≥1 NPH triad-symptom + lack of atypical clinical or neuroimaging features + not yet completed [or inconclusive response to] high-volume lumbar puncture [LP]); (3) “probable” iNPH (due to the presence of ≥1 NPH triad-symptom + presence of supportive NPH neuroimaging features + objective gait speed improvement post high-volume LP); or (4) secondary NPH (due to the presence of ≥1 NPH triad-symptom + presence of supportive NPH neuroimaging features + objective gait speed improvement post high-volume LP + presence of known risk factors for cerebral ventriculomegaly) [19]. The following were considered risk factors for secondary NPH: history of intracerebral or subarachnoid hemorrhage (SAH), significant head injury, central nervous system (CNS) infection, or presence of aqueductal stenosis [20].

Consensus meetings were held the same day to separate patients into “unlikely” NPH or “possible” NPH based on the differential neurological diagnoses considered appropriate by all involved clinicians. Patients with “possible” NPH were offered high-volume LP with pre- and post-LP gait evaluations using the 10-m walking test [21]. An interventional radiologist performed the LPs under fluoroscopy to remove a target of 50 mL of CSF. Response to high-volume LP was defined as ≥10% gait speed improvement on the 10-m walking test at least 1 h post-LP [21]. Based on the findings of the comprehensive neuropsychological assessments, patients were categorized as having a major neurocognitive disorder, mild neurocognitive disorder, or normal cognitive function. Neuropsychologists’ interpretation of the cognitive profile (subcortical vs. cortical, lateralized vs. diffuse) was used to support the differential diagnosis of NPH versus competing neurological diagnoses (e.g., a neurodegenerative process) at the consensus meetings.

### 2.3. Management Plans and Outcome

All patients finally classified as having “probable” iNPH or secondary NPH based on a positive response to high-volume LP were offered the option to undergo VPS placement [CERTAS PLUS programmable valve (Integra); initial setting programmed at 5]. Alternative management plans were considered for all other patients. These management plans were categorized into three major categories: (1) additional neurological workup; (2) referral to other specialty clinics (e.g., memory or movement disorders clinic, comprehensive spine center, geriatric medicine or geriatric psychiatry clinic); and/or (3) conservative management and rehabilitation, including intensive physical therapy, medical management of comorbidities, and follow-up with a local ± movement-disorders neurologists (See Figure 1).

Patients who underwent VPS placement were classified as responders (“shunt responsive”) or non-responders based on postoperative multidisciplinary clinical follow-up assessments at 6 and 12 months based on ≥10% gait speed improvement on the 10-m walking test and the patient’s and caregiver’s reported outcome of a functionally relevant improvement in balance and cognition. Postoperative surgical complications were monitored and shunt setting adjustments were independently made as clinically indicated (e.g., imaging findings of over or under-drainage, or if patients report gait decline after initial improvement) at neurosurgery follow-up visits at 4 weeks and 3, 6, and 12-months post-VPS placement. None of the patients was lost to follow-up.

### 2.4. Data Analysis

Group differences were tested using *t*-tests with post-hoc analyses (for continuous variables) and chi-squared tests (*X*^2^, for categorical variables).

## 3. Results

A total of 111 patients (male/female: 67/44) were evaluated during the first year of our multidisciplinary clinic. The mean age of patients was 73.2 years (±SD = 9.4). A detailed description of the entire cohort is provided in Table 1.

Based on the multidisciplinary team consensus, 52 (46.8%) were classified as “probable” iNPH. Meanwhile, 14 (12.6%) were classified as “possible” NPH, 42 (37.8%) were classified as “unlikely” NPH, and three (2.7%) were classified as secondary NPH (see Table 1). Eight of the 14 “possible” NPH patients declined a high-volume LP, primarily due to mild symptoms.

Although no significant gender differences were noted between groups of patients in the different NPH classifications, a higher number of males was observed in the “probable” iNPH group compared to other categories. Notably, patients classified as “unlikely” NPH were younger compared to “probable” iNPH or “possible” NPH (*p* < 0.05). Similarly, secondary NPH patients were younger than those in other NPH classifications (*p* < 0.0001).

The competing neurological diagnoses are detailed in Table 1. Parkinsonian syndromes were recognized in 26.2% of patients classified as “unlikely” NPH, including idiopathic PD, vascular parkinsonism, and drug-induced parkinsonism. Interestingly, 19.2% of patients with “probable” iNPH also had a comorbid movement disorder, such as essential tremor, idiopathic PD, and vascular parkinsonism. Meanwhile, AD and other dementia types (e.g., vascular or mixed type) were identified in 45.2% of the “unlikely” NPH patients (vs. 9.6% in “probable” iNPH). Other frequent neurological comorbidities noted in our cohort included spinal degenerative disorders (e.g., lumbar spinal stenosis), lumbosacral radiculopathies, and peripheral polyneuropathy, leading to further workup and intervention or intensive rehabilitation.

The most frequent gait abnormalities identified in patients with “probable” iNPH were wide-based gait (88.5%), slow gait with reduced stride length and speed (80.8%), and instability on turns (57.7%). Compared to “unlikely” NPH, wide-based gait and instability on turns were significantly more frequent in “probable” iNPH (*p* < 0.0001) and secondary NPH (*p* < 0.01). Although the median UPDRS-III scale score was higher in “probable” iNPH, no significant group differences were noted across NPH classifications (see Table 1).

Of patients deemed candidates for VPS (n = 55), 41 underwent shunt placement. The remaining patients selected conservative management (n = 8) or were excluded due to significant medical comorbidities precluding surgical intervention (n = 6). Most importantly, following the same-day consensus meeting, personalized management plans were established for all patients not considered for shunting (n = 70; see details in Table 1). Specifically, all “possible” NPH (n = 14) and remaining “probable” iNPH (n =11) patients were managed conservatively and followed in the movement disorders clinic for further work-up, medical management, and observation of symptom progression.

Of the 41 shunted patients, 87.8% (n = 36) were classified as responsive (i.e., shunt-responsive iNPH) at the 12-month follow-ups based on ≥10% gait speed improvement on the 10-m walking test and the patient’s and caregiver’s reported outcome of a functionally relevant improvement in balance and cognition. Nine patients (21.9%) had postoperative complications, including subdural hygroma/hemorrhage (n = 8) and intraparenchymal/intraventricular hemorrhage (IPH/IVH; n = 1). The patient who developed IPH/IVH required placement of a temporary external ventricular drain and a later VPS replacement. The remaining patients were managed conservatively with valve setting changes leading to complete resolution of symptoms and radiographic features of over-drainage.

## 4. Discussion

As the clinical manifestations of iNPH, including gait, locomotor, and balance disturbances, often mimic symptoms of different movement disorders, the NPH study group of the International Parkinson and Movement Disorders Society recently highlighted the role of movement disorder neurologists in the interdisciplinary evaluation of patients. Our study supports this by demonstrating the complex and diverse presentations of patients with suspected NPH and illustrating the spectrum of neurological and movement disorders that mimic or accompany iNPH in addition to AD, other dementia types, and spine disorders [1,7,8,9,10,22]. A novelty of our approach is the integration of a movement disorder neurologist into a same-day fast-track multidisciplinary NPH clinic to aid the identification of competing parkinsonian syndromes and movement disorders that need expedited attention. Our results show that incorporating a movement disorder neurologist in the fast-track evaluation is feasible and results in improved diagnostic precision, streamlined and efficient planning of further neurological workups, necessary spinal interventions, and medical management or rehabilitation.

Gait abnormality is usually the first and most bothersome symptom of iNPH [23]. Frequently described gait abnormalities in NPH include hypokinetic, broad-based walking with reduced speed and step length, hesitation, freezing of gait, and postural instability [23,24,25]. As previously highlighted, in our cohort of patients with “probable” iNPH and secondary NPH (those with a positive response to high-volume LP), wide-based gait and instability on turns appeared to be characteristic and distinguishing clinical features from “unlikely” NPH [24,26]. Similarly, parkinsonian symptoms are common in NPH patients [27]. In a population-based study, parkinsonism was identified in 71% of NPH patients. Bradykinesia and rigidity were twice, and postural instability was three times as frequent in NPH patients than in those with unlikely NPH [27]. Distinguishing the underlying etiology of parkinsonism in patients with clinical presentations suggestive of NPH can be challenging and often requires experts trained in movement disorders. Notably, in our cohort of “probable” iNPH patients, close to 20% of patients had a co-morbid movement disorder, including essential tremor, idiopathic PD, and vascular parkinsonism. The UPDRS-III scores were considerably low across groups, but a higher median value was noted for “probable” iNPH. This is comparable to a study by Molde and colleagues (2017), in which the UPDRS-III scores correlated significantly with NPH symptoms and the degree of cerebral ventriculomegaly [27]. Even though we did not identify significant UPDRS-III score differences between “probable” iNPH and “unlikely” NPH, this could be attributed to the comparable frequency of parkinsonian syndromes in each group.

To accelerate workup, we held a same-day multidisciplinary consensus meeting and ensured all patients, except those classified as “unlikely” NPH, underwent high-volume LP when clinically feasible. Based on our first-year experience, 41 (37%) patients underwent VPS placement. Of those, 87.8% were classified as “shunt-responsive”. This aligns with prior studies that showed a clinical improvement in 70–92% of shunted patients [28,29,30]. We avoided delaying care by offering alternative a priori management plans to all other patients not considered for shunt surgery. This included a combination of extensive rehabilitation, medical management of co-morbidities, and close follow-up with a movement disorders neurologist for the remaining “probable” iNPH and all “possible” NPH patients.

## 5. Limitations

The present study is limited by the lack of a detailed description of the neuropsychological findings and outcomes. We plan to analyze the comprehensive neuropsychological findings separately with a separate focus. Further, our clinical inclusion criteria required radiographic evidence of ventriculomegaly supported by an Evan’s Index > 0.3 only as established in the 2005 international iNPH guidelines [19]. We did not apply a structured radiological assessment, like the Radscale, which has proven to be a valuable diagnostic screening tool for iNPH [31]. Finally, the functional outcome of “possible” NPH and “probable” iNPH patients managed conservatively was not considered. We propose that future studies focus on comparing the functional outcomes of conservative management of iNPH to the results of shunt treatment. Our patient cohort reveals a notably high rate of individuals (20%) not proceeding with VPS treatment after being considered for surgery. We attribute this to our fast-track approach, which aims to expedite the management of patients with suspected NPH. This approach has enabled more inclusive patient recruitment and an option for clinical monitoring and rehabilitation rather than immediate surgical intervention. Based on this, our fast-track approach may introduce bias in the classification and diagnosis within this clinical cohort.

## 6. Conclusions

This study supports the complex presentations of patients with NPH and highlights the prevalence of locomotor and movement disorders that mimic or accompany NPH as relevant comorbidities. Integrating movement disorder specialists into a 1-day fast-track clinic improved diagnostic precision and efficiently streamlined care planning, including further neurological workups, necessary spinal interventions, and medical management or rehabilitation. Building on our findings and multidisciplinary experience, we recommend that future studies focus on medical and conservative management outcomes, particularly in patients not deemed VPS candidates. These results should be compared to the functional outcomes of VPS treatment in “probable” iNPH to assess competitiveness.

## Figures and Tables

**Table 1:** Demographics and Clinical Features of All Patients with Suspected NPH.

	“Probable” iNPH (n = 52)	“Possible” NPH (n = 14)	“Unlikely” NPH (n = 42)	Secondary NPH (n = 3)
Sex:				
Male	35 (67.3%)	7 (50.0%)	22 (52.4%)	3 (100%)
Female	17 (32.7%)	7 (50.0%)	20 (47.6%)	0 (0%)
Age (mean (SD)	75.4 (6.7)	75.1 (4.6)	71.0 (12.1) *	56.6 (2.5) ***
Referring Clinician				
Neurologist	28 (53.9%)	5 (35.7%)	16 (38.1%)	2 (66.7%)
Neurosurgeon	1 (1.9%)	4 (28.6%)	3 (7.1%)	1 (33.3%)
PCP	14 (26.9%)	1 (7.1%)	17 (40.5%)	0
Hospitalist/ER Physician	9 (17.3%)	4 (28.6%)	6 (14.3%)	0
Relevant Family History ^+^:				
Yes	14 (26.9%)	1 (7.1%)	10 (23.8%)	1 (33.3%)
Vascular Risk Factors:				
Hypertension	38 (73.1%)	11 (78.6%)	25 (59.5%)	2 (66.7%)
Dyslipidemia	38 (73.1%)	9 (64.2%)	19 (45.2%)	2 (66.7%)
Diabetes mellitus-type 2	17 (32.7%)	7 (50.0%)	11 (26.1%)	0
Coronary Artery Disease	14 (26.9%)	2 (14.3%)	8 (19.0%)	1 (33.3%)
Peripheral Vascular Disease	3 (5.8%)	0	0	0
Smoking History	17 (32.7%)	6 (42.9%)	12 (28.6%)	0
Risk Factors for Secondary NPH:				
Subarachnoid/Intracerebral Hemorrhage	0	1 (7.1%)	1 (2.4%)	2 (66.7%)
Head Injury	0	2 (14.2%)	7 (16.7%)	2 (66.7%)
Aqueductal Stenosis	0	0	0	0
NPH Triad Symptoms:				
Gait Disturbance	52 (100%)	13 (92.9%)	36 (85.7%)	2 (66.7%)
Cognitive Symptoms	51 (98.1%)	11 (78.6%)	38 (90.5%)	3 (100.0%)
Urinary Symptoms	47 (90.4%)	12 (85.7%)	28 (66.7%)	3 (100.0%)
Number of Triad Symptoms:				
Three	46 (88.5%)	9 (64.3%)	25 (59.5%)	2 (66.7%)
Two	6 (11.5%)	4 (28.6%)	10 (23.8%)	1 (33.3%)
One	0	1 (7.1%) ^#^	7 (16.7%) ^#^	0
Identified Gait Abnormality:				
Wide-based gait	46 (88.5%) ***	2 (14.3%)	10 (27.0%)	2 (66.7%) *
Reduced stride length/speed	42 (80.8%)	11 (78.6%)	28 (75.7%)	2 (66.7%)
Freezing of gait	5 (9.6%)	1 (7.1%)	5 (13.5%)	1 (33.3%)
Instability on turns	30 (57.7%) **	5 (35.7%)	11 (29.7%)	3 (100.0%) **
Abnormal arm swing	11 (21.2%)	5 (35.7%)	11 (29.7%)	1 (33.3%)
UPDRS-III: median (range)	18 (3–54)	13 (4–51)	12 (0–48)	6 (2–30)
Neurocognitive Diagnosis (n = 108): ^##^				
Normal	9 (17.3%)	3 (21.4%)	7 (16.7%)	0
Mild Neurocognitive Disorder	29 (55.7%)	5 (35.7%)	17 (40.5%)	3 (100%)
Major Neurocognitive Disorder	13 (25.0%)	6 (42.9%)	16 (38.1%)	0
MMSE at Baseline: median (range)	27 (8–30)	26 (9–30)	26 (4–30)	29 (22–29)
Concomitant Neurological Diagnoses:				
Movement Disorders:	10 (19.2%)	1 (7.1%)	11 (26.2%)	2 (66.6%)
Idiopathic Parkinson’s disease	3 (5.8%)	1 (7.1%)	4 (9.5%)	0
Atypical parkinsonian syndrome	0	0	2 (4.8%)	0
Vascular parkinsonism	3 (5.8%)	0	1 (2.4%)	1 (33.3%)
Drug-induced parkinsonism	0	0	3 (7.1%)	0
Essential Tremor	4 (7.7%)	0	1 (2.4%)	1 (33.3%)
Other Neurodegenerative Disorders:	5 (9.6%)	7 (50%)	19 (45.2%)	0
AD dementia	3 (5.8%)	4 (28.6%)	14 (33.3%)	0
Dementia- other subtype (e.g., vascular or mixed type)	2 (3.8%)	3 (21.4%)	5 (11.9%)	0
Spine and Peripheral Nervous System Conditions:	34 (65.4%)	10 (71.4%)	21 (50%)	0
Peripheral Neuropathy	15 (28.8%)	2 (14.3%)	5 (11.9%)	0
Myelopathy	4 (7.7%)	1 (7.1%)	4 (9.5%)	0
Lumbosacral Radiculopathy	6 (11.5%)	2 (14.3%)	2 (4.8%)	0
Lumbosacral Spondylosis with Severe Spinal Stenosis	9 (17.3%)	4 (28.6%)	2 (4.8%)	0
Other ^++^	4 (7.7%)	1 (7.1%)	8 (19.0%)	2 (66.7%)
VPS placement	41 (78.8%)	0	0	0
Management Plans:				
Additional Neurological workup:				
DAT Scan	3 (6.5%)	1 (7.1%)	5 (11.9%)	0
MRI Spine	5 (10.9%)	3 (21.4%)	1 (2.4%)	0
NCSs/EMG	4 (8.7%)	0	5 (11.9%)	0
Referral to Specialty Clinic:				
Memory Clinic	1 (1.9%)	4 (28.6%)	11 (26.2%)	0
Movement Disorders Clinic	3 (5.8%)	8 (57.1%)	13 (31.0%)	0
Geriatric Medicine/Psychiatry Clinic	1 (1.9%)	0	2 (4.7%)	0
Comprehensive Spine Center	0	2 (14.3%)	3 (7.1%)	0
Conservative Management ^###^:	11 (21.2%)	14 (100%)	24 (57.1%)	3 (100%)

DAT (dopamine transporter). NCSs: nerve conduction studies. EMG: electromyography. SD: standard deviation; * *p*< 0.05; ** *p* < 0.01; *** *p* < 0.001. ^+^ Family history of NPH, Parkinson’s disease, atypical Parkinsonian syndrome, AD, or other dementia type. ^#^ These patients only reported cognitive symptoms. ^##^ Data available for n = 108 patients (n = 3 could not complete the testing due to severe dementia). ^++^ Other: This includes traumatic brain injury, post-stroke neurological deficits, atypical parkinsonian syndrome(s) (e.g., corticobasal syndrome), and multiple sclerosis. ^###^ This included intensive physical therapy, medical management of co-morbidities, and neurologist follow-up.

## Data Availability

The data presented in this study are available on request from the corresponding author per the IRB protocol for the study.

## Supplementary Materials

The following supporting information can be downloaded at: https://www.mdpi.com/article/10.3390/jcm13206135/s1, Neuropsychological Evaluation Battery and Tests.
