# Supplementary material for: Experience from a Fast-Track Multidisciplinary Clinic Integrating Movement Disorders Neurologists in Normal Pressure Hydrocephalus Evaluation
Source: J Clin Med. 2024 Oct 15;13(20):6135. doi: 10.3390/jcm13206135 (PMC11509069; doi:10.3390/jcm13206135)
Supplement: Supplementary file 1 [file jcm-13-06135-s001.zip › jcm-3216653-supplementary.pdf]

Supplementary Material Legend: Neuropsychological Evaluation Battery and Tests

SUPPLEMENTAL MATERIAL:

Neuropsychological Evaluation Battery:

Geriatric Depression Scale (short form)
Generalized Anxiety Disorders 7-item Screen (GAD-7)
Neuropsychological Assessment Battery (Naming Subtest)
Clock Drawing Test
Purdue Pegboard (dominant, non-dominant, and bimanual)
Repeatable Battery for the Assessment of Neuropsychological Status (Line Orientation, Figure Copy, List, Story, and Figure Memory)
Mini Mental State Examination (MMSE)
Stroop Color-Word Interference (Comalli version)
Trail Making Test (Parts A & B)
Verbal Fluency (FAS, Animals)
Wechsler Adult Intelligence Scale (WAIS-IV: Digit Span Forward and Backward)
Wide Range Achievement Test-4 (WRAT-4; Reading)
